# Simultaneous or staged resection for synchronous liver metastasis and primary rectal cancer: a propensity score matching analysis

**DOI:** 10.1186/s12876-022-02250-9

**Published:** 2022-04-21

**Authors:** Elias Karam, Petru Bucur, Camille Gil, Remy Sindayigaya, Nicolas Tabchouri, Louise Barbier, Urs Pabst-Giger, Pascal Bourlier, Thierry Lecomte, Driffa Moussata, Sophie Chapet, Gilles Calais, Mehdi Ouaissi, Ephrem Salamé

**Affiliations:** 1 Colorectal Surgery Unit, Department of Digestive, Oncological, Endocrine, Hepato-Biliary, Pancreatic and Liver Transplant Surgery, Trousseau Hospital, Avenue de La République, Chambray les Tours, France; 2 EA4245 Transplantation, Immunologie, Inflammation, Université de Tours, Tours, France; 3 Department of Hepatogastroenterology and Digestive Oncology, Trousseau Hospital, Chambray les Tours, France; 4 Department of Radiotherapy, Bretonneau Hospital, Tours, France

**Keywords:** Rectal cancer, Synchronous liver metastasis, Simultaneous resection, Staged resection

## Abstract

**Background:**

Colorectal cancer is the third most common cancer in France and by the time of the diagnosis, 15–25% of patients will suffer from synchronous liver metastases. Surgery associated to neoadjuvant treatment can cure these patients, but few studies focus only on rectal cancer. This study was meant to compare the outcomes of patients who underwent a simultaneous resection to those who underwent a staged resection (rectum first or liver first) in the University Hospital of Tours, France.

**Methods:**

We assessed retrospectively a prospective maintained data base about the clinical, pathological and survival outcomes of patients who underwent a simultaneous or a staged resection in our center between 2010 and 2018. A propensity score matching was used, considering the initial characteristics of our groups.

**Results:**

There were 70 patients (55/15 males, female respectively) with median age 60 (54–68) years. After matching 48 (69%) of them underwent a staged approach and 22 (31%) a simultaneous approach were compared. After PSM, there were 22 patients in each group. No differences were found in terms of morbidity (*p* = 0.210), overall survival (*p* = 0.517) and disease-free survival (*p* = 0.691) at 3 years after matching. There were significantly less recurrences in the simultaneous group (50% vs 81.8%, *p* = 0.026).

**Conclusions:**

Simultaneous resection of the rectal primary cancer and synchronous liver metastases is safe and feasible with no difference in terms of survival.

**Supplementary Information:**

The online version contains supplementary material available at 10.1186/s12876-022-02250-9.

## Background

Colorectal cancer (CRC) is the 3rd most common cancer in France and represents about 20% of cancers [1, 2]. Upon diagnosis, 15 to 25% of patients have synchronous liver metastases (SLM). Although only 20% of CRC + SLM patients are eligible for surgical resection, a 5-year overall survival of 60% can be achieved when surgery is feasible [3]. Whether CRC and SLM resection should be performed separately or simultaneously remains controversial [4, 5]. In patients with initially resectable SLM, three strategies can be proposed. The “Conventional” or “historical” staged strategy consists in initially treating CRC then SLM resection with perioperative chemotherapy. It allows to control the evolution of CRC and decreases the risk of bowel obstruction or rectal symptoms [6–8]. The ‘‘Liver-first’’ strategy consists in initially resecting SLM with perioperative chemotherapy (CT), followed by CRC management, especially used for patients with significant metastatic liver disease and an asymptomatic primary tumor [9]. Specifically for rectal cancer, recently some authors proposed an “interval strategy”, consisting in long-course radio-chemotherapy (RT/CT), followed shortly after completion by resection of liver metastases and 6 to 8 weeks after the end of RT, rectal surgery [10]. Another possibility is the simultaneous CRC + SLM resection with promising results in well-selected patients with few co-morbidities and little extensive liver damage (mainly minor hepatectomies) [11–17]. Numerous retrospective studies have compared the simultaneous or staged strategies. Their findings have been reported in 5 recent reviews and meta-analyses [12, 18–21]. CT and RT protocols have evolved over time and laparoscopic procedures now yield similar oncologic outcomes to open procedures [22–24]. However, these studies do not differentiate between rectal and colonic primary and even though surgical techniques, with specific operating time, morbidity and mortality. Studies targeting rectal cancer [24], including mostly rectal tumors [25], or having performed specific subgroup analyses on rectal cancer [16] have shown contradictory results in the different strategies previously described. Only one prospective randomized clinical trial compared these two strategies but only 38% of included patients had rectal cancer [26]. More recent studies show a better survival for rectal cancers with SLMs compared to colon cancer with SLMs [26], safety and satisfactory oncological outcomes of simultaneous resection in both colonic and rectal cancers with SLMs [28]. Valdimarsson et al. [29] suggest simultaneous resection for colorectal cancer and SLMs show no difference in terms of survival compared to bowel first strategy, even though they experienced more complications. The aim of this study was to compare simultaneous and staged managements of rectal cancers with SLMs in terms of post-operative morbidity and mortality, recurrence, and survival and between 2010 and 2018 in the University Hospital of Tours, France.

## Methods

### Study population

Of 500 patients treated for rectal cancer at the University Hospital of Tours between January 2010 and December 2018**,** 70 patients with rectal cancer and SLM were selected. Exclusion criteria were an unachievable R0 resection (on rectum and SLMs), extra-hepatic localization, intraoperatively discovered hepatic localization, liver metastases appearing more than 6 months after rectal cancer diagnosis. SLMs were defined as all liver lesions discovered before or simultaneously with primary rectal cancer. Patients had a thoraco-abdominal CT scan and a liver magnetic resonance imaging (MRI) scan as part of the extension assessment. Rectal cancer assessment included clinical examination (digital pelvic exam, anoscopy) and paraclinical investigations (complete colonoscopy with biopsies, rectal endoscopic ultrasound (EUS), pelvic MRI). Evaluation of T stage was dependent on EUS, according to Hildebrandt et al. [30] and preoperative liver and pelvic MRI. These investigations confirmed rectal cancer location (low, 0 to 5 cm from the anal verge; mid, 5 to10; upper, 10 to 15) and assessed lymph node involvement.

### Preoperative treatment-oncologic bridge treatment (Fig. 1a, b and Additional file 1: Fig. S1)

**Fig. 1:**
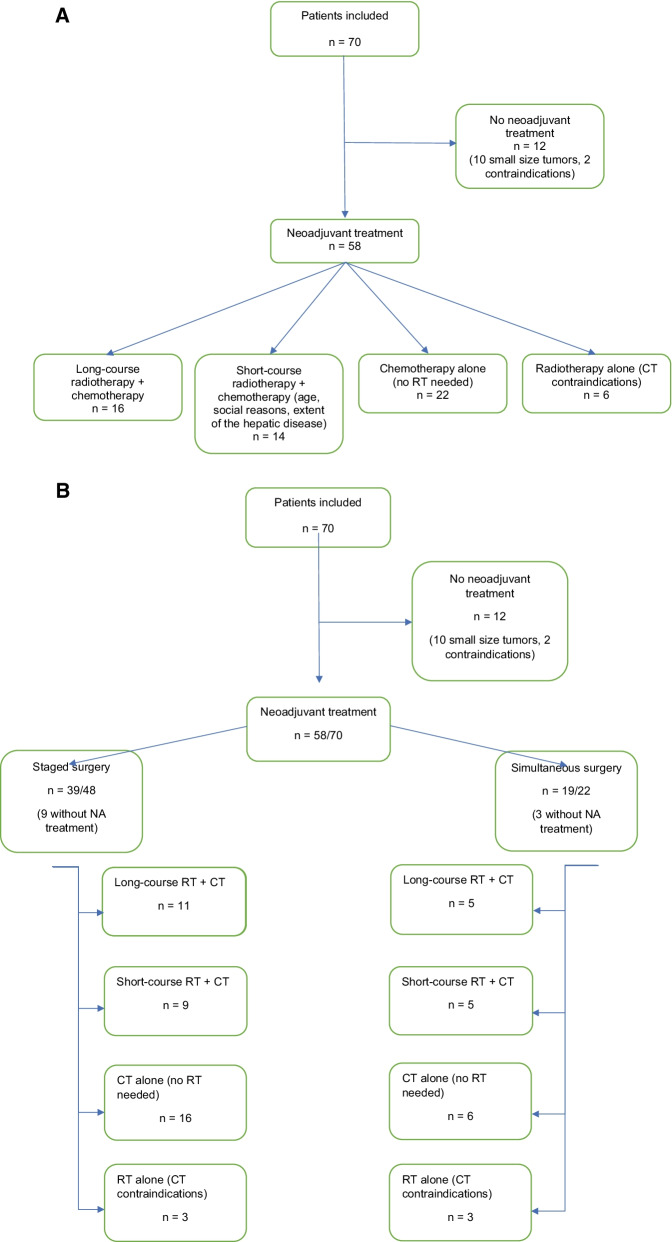
**a** Flowchart of 70 patients with rectal cancer and synchronous liver metastases according to their neoadjuvant management. **b** Comparison flowchart of patients’ neoadjuvant managements between staged and simultaneous group

5 fluorouracil or capecitabin-based Chemotherapy (CT) (5FU) were administered concomitantly with RT to 16 patients according to our guidelines for patients with mid/low rectal tumors and/or T3/4 rectal tumors. Short-course RT was chosen for 14 patients to have closely sequenced sessions because of the extent of the hepatic disease, age, or personal reasons. 22 patients underwent CT alone because of no indication for RT and six patients underwent RT alone because of CT contra-indications. Twelve patients underwent surgery without neoadjuvant treatment: ten because of small-sized rectal tumours and liver SLM, two because of cardiology-related contra-indications. Overall, 58 out of 70 patients had a neoadjuvant treatment, 39 in the staged surgery group and 19 in the simultaneous group. Twelve patients did not receive any NA treatment because of small size tumours or contraindications. Concerning the neoadjuvant treatment in each group, 5 fluorouracil or capecitabin-based chemotherapy were administered concomitantly with RT (50 Gy in 25 fractions over five weeks) according to institutional recommendations for patients with mid/low rectal tumors and/or T3/4 rectal tumors. Short-term RT was chosen because of the extent of liver disease, age, or social reasons to have closely sequenced therapy sessions. CT alone was performed when there was no need for rectal RT and RT alone was performed because of CT contraindications.

Concerning the staged surgery group, bridge treatment was CT alone for 5 patients mostly depending on SLM size, associated in six cases with a pre-operative portal embolization. Twenty patients had CT alone after second surgery, and 13 patients had CT only after both surgeries were performed, including two patients who underwent hepatic stereotaxic RT. Decision to deliver post-operative CT was based on tumor histologic type and/or surgical margins. Concerning the simultaneous surgery group, 18 patients underwent adjuvant CT, with no stereotaxic RT associated, based on tumor histologic type and/or surgical margins.

### Evaluation of tumor response

All patients had a clinical re-evaluation six weeks after the end of neo-adjuvant therapy with standard clinical examination (digital exam, anal examination). EUS was performed by the same team as the initial evaluation. Evolution of pre-treatment T and N staging was re-evaluated by pelvic MRI scan according to the 1.1 version RECIST criteria [30]. SLMs were reevaluated after neoadjuvant treatment by CT scan and MRI scan.

### Choice of surgical procedure

Patients underwent a simultaneous approach, either through laparotomy or laparoscopy, if the surgical procedure on the liver was not a major hepatectomy (< 3 segment of liver). On the contrary, if liver metastases implied a major hepatectomy (> 3 segment or required a preoperative embolization), a two-staged approach was preferred.

Moreover for the low and advanced rectal cancer requiring abdominal perineal resection or coloanal anastomosis, surgical stage approached was preferred.

### Surgical procedures

Patients underwent mechanical bowel preparation before surgery. For rectal surgery, laparoscopic medial to lateral approach was the standard approach. For T4 tumors, open approach was preferred [32]. A medial-to-lateral approach was used. Specimens were retrieved from abdominal cavity via a small abdominal incision [33]. Mechanical colorectal or manual colo-anal anastomoses (side-to-end or end-to-end) were performed depending on tumor level. Total or partial intersphincteric resection for very low rectal cancer was performed whenever feasible. Otherwise, a perineal abdominal amputation was performed. Upper rectal cancer underwent partial mesorectal excision with a 5 cm margin from the lower limit of the tumor. Other patients underwent standard TME. A protective loop ileostomy was performed routinely for mid and low rectum tumours.

Surgery was performed for curative intent. Whenever necessary, liver resection was combined with radiofrequency ablation (RFA). Major hepatectomy was defined as a liver resection comprising 3 or more contiguous liver segments. Portal vein embolization was considered if calculated remaining liver volume was insufficient (< 30% total liver volume) [34]. Two-stage hepatectomy involved initial resection of all left liver metastases with ligation of the right portal vein or embolization of the right portal vein in the postoperative period. Four to 6 weeks after first surgery, a right or extended hepatectomy was performed. Liver transection was carried out using squeeze-clamp technique and ultrasound dissection to expose residual vessels or glissory sheaths, which were ligated with 4–0 vicryl or sealed using LigaSure technology [34–37]. Intraoperative ultrasonography was routinely performed to guide SLM resection.

### Pathology results

Primary tumors were analyzed using a standardized protocol [37]. TNM classification was used according to the 8^th^ edition of American Joint Committee of Cancer (AJCC). Circumferential and distal resection margins were defined as positive (R +) if less than 1 mm and negative (R0) if more than 1 mm to the tumor.. Total or partial mesorectal excision, colloid component degree, differentiation grade, vascular, lymphatic or perinervous emboli, liver fibrosis, liver steatosis and capillary obstruction syndrome were stated in pathology report.

### Short-term outcomes

Anastomotic leakage was defined and graded according to the International Study Group of Rectal Cancer [39]. Any clinical (sepsis, peritonitis, emission of gas, pus, or feces from the pelvic drain, purulent discharge per anus, or rectovaginal fistula) and/or biological suspicion of AL led to an early CT-scan. Management included antibiotics, radiologic or transanal drainage, and/or early abdominal redo surgery [40]. Patients presenting with postoperative bile leakage were treated with antibiotics and radiological or surgical drainage [41]. Short-term 30-day postoperative complications were ranked according to Clavien Classification [42].

### Long-term outcomes

Recurrence-free and overall survivals were analyzed. Postoperative follow-up included clinical, biochemical, and radiological assessments every three months during first postoperative year, then every six months up to five years postoperatively and every year up to ten years. Surviving patients were assessed for disease recurrence and site of recurrence. Follow-up data were obtained from medical records and direct patients’ consultation.

### Statistical analysis

Propensity score matching (PSM) using nearest-neighbor method was performed to match patient who underwent simultaneous resection of liver metastasis to those who underwent staged procedures. PSM model was generated using preoperative risk factors reported to impair patient’s survival, namely, surgical difficulty (i.e., minor, or major hepatectomy) and underlying liver fibrosis. Qualitative variables are presented in percentages and compared using χ2 test with Bonferroni correction whenever necessary. Quantitative variables are presented in medians and interquartile range and compared using Student or Wilcoxon test. Kaplan–Meier method was used to estimate recurrence-free survival and overall survival, which were compared using the Log-rank test. Continuous variables were compared using ANOVA or nonparametric ANOVA tests. This study was conducted according to the ethical standards of the Committee on Human Experimentation of our institution and reported according to the Strengthening the Reporting of Observational Studies in Epidemiology (STROBE) guidelines [43].

## Results

Out of 70 patients with rectal cancer and SLMs, 48 were in the staged surgery group and 22 in the simultaneous surgery group. After PSM, there were 22 patients in each group.

### Demographic and pre-operative characteristics (Table 1)

**Table 1:** Demographic and preoperative characteristics of the 70 patients with rectal cancer and synchronous liver metastases according to their surgical management

	Before matching				After matching		
	Overall population	Staged surgery	Simultaneous surgery	*P*	Staged surgery	Simultaneous surgery	*P*
N (%)	70 (100)	48 (69)	22 (31)		22 (50)	22 (50)	
*Age (years), median* ± *IQR*	64 (54–68)	61 (54–67)	65 (60–77)	0.0644	59 (52–65)	65 (60–77)	**0.012**
≥ 60 years, n (%)	46 (65.7)	28 (58.3)	18 (81.8)	0.0634	11 (50)	18 (81.8)	**0.026**
< 60 years, n (%)	24 (34.3)	20 (41.7)	4 (18.2)		11 (50)	4 (18.2)	
Sex ratio (female/male)	15/55	13/35	2/20	0.1208	8/14	2/20	**0.021**
BMI (kg/m^2^), median ± IQR	25.4 (23–28)	25.6 (23–28.8)	24.8 (23.4–27.2)	**0.0244**	24.4 (22.9–26.7)	24.8 (23.2–27.5)	0.740
*ASA score, n (%)*							
1	25 (35.7)	14 (29.2)	11 (50)	0.2342	8 (36.4)	9 (40.9)	0.155
2	40 (57.2)	30 (62.5)	10 (45.5)		14 (63.6)	10 (45.5)	
3	5 (7.1)	4 (8.3)	1 (4.5)		0	3 (13.6)	
Arteriopathy, n (%)	2 (2.9)	2 (4.2)	0	1.0000	1 (4.6)	0	0.312
Diabetes, n (%)	11 (15.7)	9 (18.8)	2 (2.9)	0.4825	3 (13.6)	2 (9.1)	0.635
*Tumor diagnosis, n (%)*							
Bleeding	38 (54.2)	23 (48)	15 (68.1)	0.1300	10 (45.5)	15 (68.2)	0.128
Weight loss	11 (15.7)	6 (12.5)	5 (22.7)	0,3035	4 (18.2)	5 (22.7)	0.709
*Rectal adenocarcinoma location, n (%)*							
Upper (10–15 cm)	21 (30)	14 (29.2)	7 (31.8)	0,6572	3 (13.6)	6 (27.3)	0.449
Mid (5–10 cm)	28 (40)	18 (37.5)	10 (45.5)		10 (45.5)	10 (45.5)	
Low (2–5 cm)	21 (30)	16 (33.3)	5 (22.7)		9 (40.9)	6 (27.3)	
*T stage, n (%)*							
T1	5 (7.1)	2 (4.2)	3 (13.7)	0.4345	0	3 (13.6)	0.283
T2	6 (8.6)	5 (10.4)	1 (4.5)		2 (9.1)	1 (4.6)	
T3	51 (72.9)	36 (75)	15 (68.1)		18 (81.8)	15 (68.2)	
T4	8 (11.4)	5 (10.4)	3 (13.7)		2 (9.1)	3 (13.6)	
*Liver metastasis*							
Bilobar (n, %)	34 (48.6)	25 (35.7)	9 (41)	0.4442	13 (59.1)	9 (40.9)	0.228
Number patients > 3 metastasis	19 (27.1)	15 (31.3)	4 (18.1)	0.3860	7 (31.8)	4 (18.2)	0.296
Number of metastasis (median, IQR)	2 (1–4)	3 (1–4)	2 (1–2)	0.7100	3 (1–4)	2 (1–3)	0.226
Neoadjuvant chemotherapy, n (%)	52 (74.3)	36 (75)	16 (72.7)	1.0000	17 (77.3)	19 (86.4)	0.434
Portal embolization, n (%)	6 (8.6)	3 (6.3)	3 (13.6)	0.3699	1 (4.6)	3 (13.6)	0.294
*Neoadjuvant radiation therapy, n (%)*							
Long-course radiotherapy *	16 (22.9)	11 (23)	5 (22.7)	1.0000	5 (22.7)	5 (22.7)	0.999
Short-course radiotherapy **	14 (20)	9 (18.8)	5 (22.7)		5 (22.7)	5 (22.7)	

Number in bold correspond to an stastical difference (*P* < 0.05)

*IQR* interquartile range

*With chemotherapy

**Without chemotherapy

Seventy patients with rectal cancer and SLM were included, 48 (69%) underwent a staged procedure and 22 (31%) a simultaneous procedure. There were more male patients (sex-ratio: 15/55) with median age of 60 (54–68). Upon diagnosis, rectal tumours were mainly T3 (72.9%) with bilobar liver metastasis in 48.6% of patients and a median number of 2 metastases. There was no difference between each group regarding medical history, except BMI which was significantly higher in the staged surgery group (median 25.6 with *p* = 0.0244). Overall, 74.3% of patients underwent CT, 22.9% long-course RT and 20% short-course RT, with no differences between both groups (NS). After PSM, patients who underwent simultaneous liver resection were significantly older than those who underwent staged procedures (65 (60–77) years old *vs.* 59 (52–65) years old; *p* = 0.012). After PSM, patients who underwent simultaneous liver resection there were significantly more male than staged group (80% vs 57.1%; *p* = 0.021).

### Intra-operative parameters (Table 2 and Additional file 2: Table S1)

**Table 2:** Intraoperative parameters characteristics of the 70 patients with rectal cancer and synchronous liver metastases according to their surgical management

	Before matching				After matching		
	Overall Population	Staged surgery	Simultaneous surgery	*P*	Staged surgery	Simultaneous surgery	*P*
Number of patients	70	48 (69)	22 (31)	–	22	22	–
All procedure by laparoscopy, n (%)	9 (12.9)	2 (4.2)	7 (31.8)	**0.0032**	7 (31.8)	7 (31.8)	0.999
Laparotomy, n (%)	36 (51.4)	27 (56.2)	9 (41)	0.3050	6 (27.3)	6 (27.3)	0.999
Mixed*, n (%)	25 (35.7)	19 (39.6)	6 (27.2)	0.4229	4 (18.2)	0	**0.040**
Conversion to laparotomy, n (%)	8/34 (23.5)	6/21 (28.6)	2/13 (15.4)	0.4438	5 (22.7)	9 (40.9)	0.195
Complete TME, n (%)	47 (67.1)	35 (72.9)	12 (54.5)	0.1721	18 (81.8)	12 (54.6)	0.052
Partial TME, n (%)	23 (32.9)	13 (27.1)	10 (45.5)		4 (18.2)	10 (45.4)	
Abdominoperineal resection, n (%)	16 (22.9)	11 (22.9)	5 (22.7)	1.000	5 (22.7)	5 (22.7)	0.999
*Anastomosis technique, n (%)*							
Mechanical (stapled)	51/54 (94.4)	35/37 (94.6)	16/17	1.000	21 (95.5)	20 (90.9)	0.549
Manual	3/54 (5.6)	2/37 (5.4)	1/17		1 (4.5)	2 (9.1)	
*Liver surgery, n (%)*							
Minor hepatectomy	45 (64.3)	24 (50)	21 (95.5)	**0.0001**	21 (95.5)	20 (90.9)	0.549
Major hepatectomy	25 (35.7)	24 (50)	1 (4.5)		1 (4.5)	2 (9.1)	
Associated radiofrequency ablation	7 (10)	7 (14.6)	0	0.0893	2 (9.1)	1 (4.5)	0.549
Duration of Pringle maneuver (minutes), median ± IQR	30.5 (19–45)	30 (25–45)	13 (11–18)	0.1409	25 (0–30)	13 (0–63)	0.999
Diverting stoma, n (%)	34 (48.6)	21 (43.8)	13 (59.1)	0.3050	7 (31.8)	12 (27.3)	0.128
Delay between first and second surgery (months), median ± IQR	6 (4–8)	6 (4–8)	–	–	2 (0–5)	–	

Number in bold correspond to an stastical difference (*P* < 0.05)

IQR: interquartile range

Major hepatectomy: resection of 3 segments or more

Hybrid: laparoscopy and laparotomy

For all series, 51.4%, 12.9% and 35.7% of patients underwent all laparotomy, all laparoscopic, and mixed procedures, respectively. Conversion to laparotomy occurred overall in 23.5% because of excessive bleeding or difficulties performing dissection. Complete TME was performed in 67.1% of patients and abdominoperineal resection (APR) in 22.9% (NS). Anastomoses were mainly mechanical using staples (94.4%). Pringle manoeuvre lasted a median time of 30.5 (19–45) minutes and there were 34% of diverting loop ileostomas, with no differences between both groups. In contrast, all laparoscopy procedures were significantly higher in the simultaneous surgery group (31.8% vs 4.2%, *p* = 0.0032). There were more major hepatectomies in the staged surgery group (50% vs 4.5%, *p* = 0.0001) and more minor hepatectomies in the simultaneous surgery group (95.5% vs 50%, *p* = 0.0001). In the staged surgery group, rectum first (RF) and liver first (LF) procedures were performed in 37 (77%) and 11 (23%) patients. After PSM, patients in the “staged” group were more likely to undergo mixed procedures (18.2% vs. 0%; *p* = 0.040).

Rectum first (RF) and liver first (LF) procedures were analysed separately in the staged surgery group (Additional file 2: Table S1). There were no differences concerning the surgical approach even though there were zero laparoscopy procedures in the LF group. Conversion rate, complete TME rate, APR rate were similar, anastomosis was mechanical in 73% and 72.7% concerning respectively RF and LF groups (NS). There were significantly more major hepatectomies in the RF group (64.9% vs 0%, *p* = 0.0002) and more minor hepatectomies in the LF group compared to the RF group (100% vs 35.1%, *p* = 0.0002). No differences were found for the duration of the Pringle manoeuvre nor the diverting stoma rate, and there was a six-month median period between rectal and liver procedures.

### Post-operative outcomes (Table 3)

**Table 3:** Postoperative parameters of the 70 patients with rectal cancer and synchronous liver metastases according to their management

	Before matching				After matching		
	Overall Population	Staged surgery	Simultaneous surgery	*P*	Staged surgery	Simultaneous surgery	*P*
N (%)	70 (100)	48 (69)	22 (31)	-	22	22	
Complications	43 (61.4)	34 (70.8)	9 (40.1)	**0.0329**	16 (72.7)	12 (54.6)	0.210
Clavien I–II	24 (34.3)	18 (37.5)	6 (27.2)	0.5882	15 (68.2)	10 (45.6)	0.128
Clavien III–IV	19 (27.1)	16 (33.3)	3 (13.6)	0.1462	1 (4.6)	2 (9.1)	0.549
Sepsis, n (%)	17 (24.3)	15 (31.3)	2 (9.1)	**0.0398**	7 (31.8)	6(27.3)	0.741
Anastomotic fistula, n (%)	17 (24.3)	15 (31.3)	2 (9.1)	**0.0398**	2 (9.1)	2 (9.1)	0.999
Antibiotics only, n (%)	5 (7.1)	4 (8.3)	1 (4.5)	1.000	2 (9.1)	2 (9.1)	0.999
CT scan Drainage for colorectal complications	8 (11.4)	8 (16.7)	0	**0.0498**	0	0	**-**
Surgical drainage laparotomy for colorectal complications	4 (5.7)	3 (6.3)	1 (4.5)	1.000	0	0	-
Hepatic failure, n (%)	0	0	0	1.000	0	0	-
Biloma, n (%)	4 (5.7)	4 (8.3)	0	0.3008	2 (9.1)	0	0.148
Biloma CT-guided drainage, n (%)	3 (4.3)	3 (6.3)	0	0.5467	0	0	-
Biloma surgical drainage, n (%)	1 (1.4)	1 (2.1)	0	1.000	1 (4.6)	0	0.312
Pulmonary complications, n (%)	5 (7.1)	3 (6.3)	2 (9.1)	0.6463	0	2	0.148
Neurological complications, n (%)	3 (4.3)	2 (4.2)	1 (4.5)	1.000	0	1 (4.6)	0.311
Ileus, n (%)	9 (12.9)	6 (12.5)	3 (13.6)	1.000	1 (4.6)	3 (13.6)	0.294
Hospital stay (days), median	21 (15–29)	25 (18–30)*	12 (9–19)	**0.0249**	8 (6–15)	12 (8–28)	0.298

Post-operative hepatic failure defined as bilirubine > 50 micromol/L and prothrombine < 50%

Number in bold correspond to an stastical difference (*P* < 0.05)

*IQR* interquartile range, *CT* computed tomography

*Hospitalization stay days included two hospitalization of the two staged

The overall complications rate was 45.7% with significantly more complications in the staged surgery group (70.8% vs 40.1%, *p* = 0.0329). For the staged procedures, complications in the first and second hospital stay were added. Complications stages I-II occurred in 34.3% and stages III-IV in 27.1% of the patients. There were significantly more sepsis complications in the staged surgery group (all linked to anastomotic fistulas) 31.3% vs 9.1% (*p* = 0.0398), more CT scan drainage in the staged surgery group (16.7% vs 0%, *p* = 0.0498) because of anastomotic fistulas. There were significantly more CT scan drainages in the staged surgery group (16.7% vs 0%, *p* = 0.0498) of peri-anastomotic collections, but also higher percentages of surgical drainages for fistulas, fistulas treated with only antibiotics and bilomas (with CT-guided or surgical drainages), even though those were not significantThere were also more fistula surgical drainages, bilomas, CT and surgical bilomas drainages in the staged surgery group- However, these differences were not statistically significant different. There was no post-operative hepatic failure. The hospital stay was significantly higher in the staged surgery group with a median of 25 days vs 12 days (*p* = 0.0249). After PSM, there was no difference regarding postoperative complications rate or hospital length of stay between both groups.

### Pathology results (Table 4)

**Table 4:** Pathology results of the 70 patients with rectal cancer and synchronous liver metastases according to their management

	Before matching				After matching		
	Overall Population	Staged surgery	Simultaneous surgery	*P*	Staged surgery	Simultaneous resection	*P*
N (%)	70 (100)	48 (69)	22 (31)	–	22 (50)	22 (50)	
*Rectal pT stage, n (%)*							
pT0 pT1 pT2 pT3 pT4	5 (7.1) 3 (4.4) 5 (7.1) 49 (70) 8 (11.4)	3 (6.3) 3 (6.3) 4 (8.2) 32 (66.7) 6 (12.5)	2 (9.1) 0 1 (4.5) 17 (77.3) 2(9.1)	0.691	2 (9.1) 0 2 (9.1) 17 (77.3) 1 (4.6)	2 (9.1) 1 (4.6) 1 (4.6) 16 (72.7) 2 (9.1)	0.791
*Rectal pN stage, n (%)*							
pN0 pN1 pN2	23 (32.9) 32 (45.7) 15 (21.4)	15 (31.3) 21 (43.7) 12 (25)	8 (36.3) 11 (50) 3 (13.7)		11 (50) 8 (36.4) 3 (13.6)	9 (40.9) 10 (45.5) 3 (13.6)	0.810
Number of lymph nodes harvested, median ± IQR	25 (17–33)	24 (18–38)	26 (15–32)	0.803	27 (20–47)	26 (14–32)	0.423
Number of lymph nodes metastasis, median ± IQR	1 (0–4)	1 (0–4)	1 (0–2)	0.335	2 (0–4)	1 (0–2)	0.696
Rectal resection positive margin, n (%)	8 (11.4)	6 (12.5)	2 (9.1)	1.000	1 (4.6)	2 (9.1)	0.549
Number of liver metastasis, median ± IQR	2 (1–4)	2 (1–6)	2 (1–3)	0.207	3 (2–6)	5 (3–6)	0.453
Liver resection positive margin, n (%)	17 (24.3)	13 (27.1)	4 (18.1)	0.553	4 (18.2)	0	**0.040**
Percentage of liver metastasis necrosis, median ± IQR	50 (23–68)	50 (25–70)	45 (4–50)	0.297	40 (25–90)	25 (20–90)	0.789
Liver fibrosis, n (%)	16 (22.9)	14 (29.2)	2 (9.1)	0.074	1 (4.6)	2 (9.1)	0.550
Liver steatosis, n (%)	23 (32.9)	16 (33.3)	7 (31.8)	1.000	2 (9.1)	7 (31.8)	0.062
Capillary obstruction syndrome, n (%)	16 (22.9)	9 (18.8)	7 (31.8)	0.227	0	2 (9.1)	0.148
Largest size of liver metastasis (mm), median ± IQR	30 (18–40)	35 (18–40)	24 (15–31)	0.179	20 (10–39)	24 (16–41)	0.392
Molecular biology, n (%)							
KRAS mutation	14(20)	8 (18.8)	5 (22.7)	0.529	3 (13.6)	5 (22.7)	0.698
BRAF mutation	1 (1.4)	1 (2.1)	0	1.000	0	0	1.000
Microsatellite instability (MSI)	1/55 (1.8)	1/39 (2.5)	0	1.000	1/18 (5)	0	0.450
Unknown statu of microsatellite instability	15 (21.4)	9 (18.3)	6 (27.3)	0.532	4 (18.2)	6 (27.3)	0.720

Number in bold correspond to an stastical difference (*P* < 0.05)

*IQR* interquartile range

Rectal tumours were mainly T3 (70%) and N1 (45.7%) with medians of 25 (17–33) harvested lymph nodes and 1 lymph node metastasis. There was a median of two liver metastases resected, rectal and liver margins were positive in 11.4% and 24.3% respectively. In the staged surgery group, there was more liver fibrosis, more liver steatosis and more capillary obstruction syndrome compared to simultaneous group with no significative difference. There were no significant differences regarding the number of harvested lymph nodes or the number of metastatic lymph nodes or the number of positive rectal margins. Concerning molecular biology KRAS, BRAF mutation were observed in 20% and 1.4% of patients without statistical difference between two groups staged and simultaneous groups (13.6 vs 22.7%, 2.1% vs 0%, *p* = 0.569; *p* = 1.000). For microsatellite instability only one patient was MSI (1.8%), and was in staged group.

Concerning liver pathology, after PSM patients in the “staged” group had more often positive liver resection margins (18.2% *vs.* 0%; *p* = 0.040).

Rectum first (RF) and liver first (LF) results were analysed separately in the staged surgery group (Additional file 3: Table S2). Tumours were T3 in 67.6% of the RF and 63.6% of the LF procedures and N1 in 43.2% of the RF and 45.4% of the LF procedures. There were more R1 liver resections in contact with the liver tissue in the RF group (77.8% vs 75%), less R1 rectal resections on the lateral margin in the RF group (50% vs 100%), more liver fibrosis and steatosis in the RF group (35.1% vs respectively 9.1% and 18.2%), less capillary obstruction syndrome in the RF group (16.2% vs 27.3%), none of these results were significant. The median size of the liver metastasis was higher in the RF group but not significantly (35 vs 21 mm).

### Late postoperative and survival outcomes (Table 5, Fig. 2a, b)

**Table 5:** Late postoperative outcomes of the 70 patients with rectal cancer and synchronous liver metastases according to their management

	Before matching				After matching		
	Overall Population	Staged surgery	Simultaneous resection	*P*	Staged surgery	Simultaneous resection	*P*
N (%)	70 (100)	48 (69)	22 (31)	-	22 (50)	22 (50)	-
*Oncological results*							
Adjuvant chemotherapy, n (%)	56 (80)	38 (79.1)	18 (32.1)	1.000	7 (31.8)	6 (27.3)	0.741
Recurrence, n (%)	51 (72.9)	40 (83.3)	11 (50)	**0.0078**	18 (81.8)	11 (50)	**0.026**
Rectal	5 (7.1)	5 (10.4)	0	0.1727	2 (9.1)	0	0.148
Liver	32 (45.7)	26 (54.2)	6 (27.2)	**0.0425**	14 (63.6)	8 (36.6)	0.070
Carcinosis	11 (15.7)	8 (16.7)	3 (13.6)	1.000	2 (9.1)	3 (13.6)	0.635
*Number of recurrences sites, n (%)*							
1	36 (51.4)	26 (54.2)	10 (45.5)	0.5604	16 (88.9)	10 (90.9)	0.862
2	8 (11.4)	7 (14.6)	1 (4.5)		2 (11.1)	1 (9.1)	
3	1 (1.4)	1 (2.1)	0		0	0	
Time to recurrence (month), median ± IQR	15 (11–20)	17 (11–21)	13 (11–14)	0.3568	13 (6–25)	8 (5–11)	0.465
Median of follow up (months) ± IQR	28 (18–39)	28 (19–42)	20 (13–34)	0.0990	28 (16–38)	16 (8–38)	0.285
Overall survival at 3 years (%)	86%	85%	42%	0.0749	68%	62%	0.517
Overall survival at 5 years (%)	44%	77%	54%		45%	46%	
Disease free survival at 3 years (%)	20%	19%	4.5%	0.6851	7%	10%	0.691
Disease free survival at 5 years (%)	3.5%	24%	0%		–	–	
*Recurrence treatment, n (%)*							
Surgery	11 (15.7)	9 (18.8)	2 (9.1)	0.2998	6 (27.3)	2 (9.1)	0.118
Chemotherapy	39 (55.7)	31 (64.6)	8 (36.4)	0.7063	13 (59.1)	8 (36.4)	0.131
Radiotherapy	11 (15.7)	7 (14.6)	4 (18.2)	0.2220	3 (13.6)	4 (18.2)	0.680
Delayed fistula, n (%)	2 (2.9)	0	2 (9.1)	**0.0431**	0	0	**-**

Number in bold correspond to an stastical difference (*P* < 0.05)

*IQR* interquartile range

**Fig. 2:**
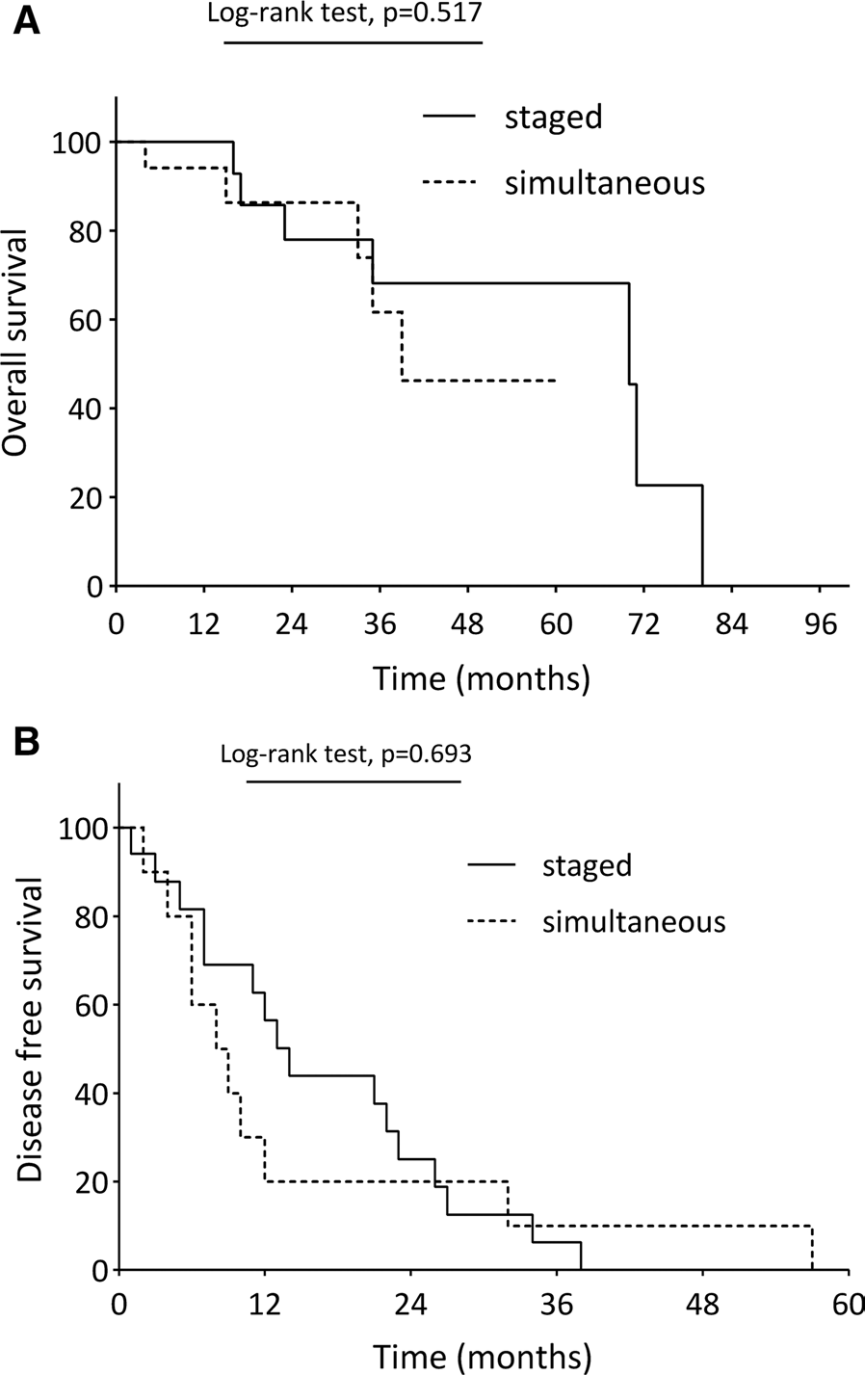
**a** Comparison of overall survival between staged surgery group (solid line) and simultaneous surgery group (dashed line). Staged surgery group (solid line) and simultaneous surgery group (dashed line). Comparaison of overall survival in staged and simultaneous surgery. X axis: time (months). Y axis: percentage surv. **b** Comparison of disease-free survival between staged surgery group (solid line) and simultaneous surgery group (dashed line). Staged surgery group (solid line) and simultaneous surgery group (dashed line). Comparison of disease free survivals between staged and simultaneous surgery groups. X axis: time (months). Y axis: percentage survival.

Median follow-up was 28 months (19–42) overall. After propensity score matching, recurrences were significantly higher in the staged surgery group (81.8% *vs.* 50%; *p* = 0.026) and tended to occur mainly in the liver (63.6% vs. 36.6%, *p* = 0.070). Recurrence occurred mainly in 1 site (51.4%) with a 15-month median period until recurrence. Recurrences were mostly treated with chemotherapy (55.7%). After PSM, overall survival at 3 and 5 years as well as disease free survival at 3 years were comparable between the “staged” and “simultaneous” groups (Fig. 2a, b).

## Discussion

Our current data show, according to propensity score matching (PSM) analysis, no difference in morbidity and survival between synchronous and staged resection of rectal cancer with synchronous liver metastasis (SLM). However, in our case series, we found a significantly higher recurrence rate in patients who underwent staged therapy.

Modern therapeutic approach for rectal cancer with resectable SLM combines rectal surgery (TME) with neoadjuvant RT or RCT and liver surgery with perioperative CT in complex therapeutic sequences. Any complication at any level of therapy can delay the therapy schedule of such a complex combined, interdisciplinary therapy approach. Primary colonic cancers do not always require locoregional neoadjuvant treatment (i.e. Radiochemotherapy, i.e. radiotherapy) as locally advanced rectal cancer. The therapy of primary rectal cancer has a higher morbidity compared to data from retrospective series on the treatment of colorectal carcinoma with SLM [37, 44]. It is reasonable to assume that combining RT and total mesorectal excision with liver surgery can increase the complication rate and long-term survival may thus be impaired.

In the current study, 70 patients were managed at a tertiary centre of expertise for colorectal and liver surgery and clinical outcomes were investigated. Our groups were not initially comparable as there were large differences between the group characteristics. One of these main differences was in the choice of surgical approach. Since we opted for a staged approach if the liver resection was expected to be extensive, we had in the group of patients who had simultaneous rectal and liver surgery, significantly fewer major hepatectomies. To address this issue, we performed a PSM analysis. We found that the patients in the staged surgery group were older but that there were no other significant differences.

Before matching, morbidity rate was higher in the staged surgery group, most likely due to the higher rate of major liver resections (three or more segments) as described previously in the literature [45]. However length of stay was shorter in this case, but again after PSM, morbidity rates and length of stay were comparable in both groups.

Ghiasloo et al. [46] observed a higher morbidity rate after a staged surgical approach, especially when primary rectal cancer was operated on first ("rectum first"). However, this large retrospective case series included not only rectal cancer patients but also colon cancer patients. Since no subgroup analysis was carried out with regard to rectal cancer patients, the significance of this study with regard to rectal cancer patients is limited. In the recently published study by Abelson et al. from 2017 [15], subgroup analysis of rectal cancer patients and SLM (New York State Department of Health Statewide Planning and Research Cooperative System database) showed no significant perioperative outcome differences between simultaneous or staged therapy with respect to important perioperative complications such as anastomotic insufficiency. Furthermore, it was shown that patients who underwent simultaneous resection had a significantly lower risk of prolonged hospitalization (OR = 0.25; 95% CI = 0.14–0.45) and high hospital costs (OR = 0.26; 95% CI = 0.14–0.45).

In 2015, Silberhumer et al. [14] reported no differences in morbidity and mortality—regardless regardless of whether rectal carcinoma was operated on first or a simultaneous rectal and SLM operation was performed. No difference was also found whether only limited or extended liver resection had to be performed.

In the only randomised trial comparing the staged and simultaneous approaches to colorectal cancer and SLMs [26], there was no difference between the two approaches in terms of morbidity. In particular there was no difference between patients who had at least one severe complication (Clavien-Dindo stages III to IV). The rate of patients who had at least one serious complication was 18% for rectal resection and 21.7% in the case of simultaneous rectal and SLM resection. However, they did not differentiate between they did not differentiate between the extent of liver resections and their staged surgery group included only initial rectal procedures.

In our current study on this, we found more positive resection margins on the liver and a higher recurrence rate in the staged surgery group after PSM analysis. Survival was not affected; however, consequences may happen in a larger cohort. Perhaps staged resection was used when more complex liver resections were needed, leading to a worse surgical clearance of the tumour.

With regard to overall survival and tumour recurrence rate, we could not detect any difference after the PSM analysis. Several large cohort studies reported the same overall and disease-free survival regardless of whether the resection was simultaneous or two-stage, and whether liver or bowel were operated on first [14, 35, 46]. In contrast, two studies reported better overall survival for the two-stage approach. The multivariate analysis in these two retrospective studies found that simultaneous resection was not a risk factor for worse prognosis, although patients in the staged treatment received significantly more chemotherapy (CT) treatments. [14, 35]. In contrast, Slupski et al. [47], found patients in the simultaneous resection group having a better overall survival. This result could be explained by a significantly higher burden of disease in patients undergoing a two-stage approach. For De Haas et al. [36], simultaneous resection of colorectal carcinomas and SLMs had a lower morbidity rate than a two-stage resection. However, the simultaneous approach was also shown in a multivariate analysis to be an independent predictor of tumour recurrence three years later.

## Conclusion

Our results appear to be consistent with the existing literature and confirm the feasibility and safety of simultaneous resection of rectal cancer with SLM. Furthermore, survival did not differ between the two groups, although the positive resection margins and recurrence rate were higher in the staged surgery group. Our results need to be confirmed by other large case series. To the best of our knowledge, the present case series is one of the first to focus exclusively on rectal cancer with SLMs. Limitations of this study include the retrospective study design, initially non-comparable groups, which we compensated for using the PSM analysis and an alternating therapeutic approach with a shift from conventional open or to laparoscopic surgery during the study period. However, these weaknesses reflect the natural evolution of standard of care over time and are also a reflection of the clinical practice. Simultaneous resection of rectal primary cancer and synchronous liver metastases is safe and feasible with no difference in terms of survival.

## Supplementary Information

**Additional file 1: Figure S1**. Comparison flowchart of patients’ adjuvant managements between staged and simultaneous group.

**Additional file 2**. **Table S1.** Intraoperative parameters of the 48 patients with rectal cancer and synchronous liver metastases undergoing staged surgery according to their management.

**Additional file 3**. **Table S2.** Pathology results of the 48 patients with rectal cancer and synchronous liver metastases undergoing staged surgery according to their management.

## Data Availability

The datasets used and analyzed during the current study are available from the corresponding author on reasonable request.

## Abbreviations

CRC: Colorectal Cancer
SLM: Synchronous liver metastasis
MRI: Magnetic resonance imaging
CT: Cumputed tomography
RCT: Radio chemo therapy
RT: Radiotherapy
CT: Chemotherapy
NA: Neoadjuvant
TME: Total Mesorectal Excision
ASA: American Society of Anesthesiology score
EUS: Endorectal Ultrasound
RFA: Radio frequency ablation
APR: Abdominoperineal resection
RF: Rectum first
LF: Liver first
PSM: Propensity score matching
