# Supplementary material for: Simultaneous or staged resection for synchronous liver metastasis and primary rectal cancer: a propensity score matching analysis
Source: BMC Gastroenterol. 2022 Apr 21;22:201. doi: 10.1186/s12876-022-02250-9 (PMC9026992; doi:10.1186/s12876-022-02250-9)
Supplement: Supplementary file 1 — Additional file 1: Figure S1. Comparison flowchart of patients’ adjuvant managements between staged and simultaneous group. [file 12876_2022_2250_MOESM1_ESM.docx]

|  |  |  |  |  |  |  |  |  |  |
| --- | --- | --- | --- | --- | --- | --- | --- | --- | --- |
|  |  |  |  |  |  |  |  |  |  |

Patients included

n = 70

Figure 1S: Comparison flowchart of patients’ adjuvant managements between staged and simultaneous group

CT after both surgeries (including two hepatic stereotaxic RT)

n = 13

Adjuvant CT (no RT associated)

n = 18

CT only after 1^st^ surgery

n = 5

CT only after 2^nd^ surgery (no RT associated)

n = 20

Simultaneous surgery

n = 18/22

Staged surgery

n = 38/48

Adjuvant treatment

n = 56/70

Patients included

n = 70
