# Supplementary material for: Simultaneous or staged resection for synchronous liver metastasis and primary rectal cancer: a propensity score matching analysis
Source: BMC Gastroenterol. 2022 Apr 21;22:201. doi: 10.1186/s12876-022-02250-9 (PMC9026992; doi:10.1186/s12876-022-02250-9)
Supplement: Supplementary file 2 — Additional file 2. Table S1. Intraoperative parameters of the 48 patients with rectal cancer and synchronous liver metastases undergoing staged surgery according to their management. [file 12876_2022_2250_MOESM2_ESM.docx]

Table S1: Intraoperative parameters of the 48 patients with rectal cancer and synchronous liver metastases undergoing staged surgery according to their management

|  | Rectum first surgery | Liver first surgery | P |
| --- | --- | --- | --- |
|  |  |  |  |
| Number of procedures | 37 | 11 |  |
| Laparoscopy, n (%) | 2 (5.4) | 0 | 0.4006 |
| Laparotomy, n (%) | 19 (51.4) | 8 (72.7) |  |
| Mixed*, n (%) | 16 (43.2) | 3 (27.3) |  |
| Conversion to laparotomy, n (%) | 5/18 (27.8) | 1/3 (33.3) | 0.6220 |
| Complete TME, n (%) | 27 (73) | 8 (72.7) | 0.9872 |
| Partial TME, n (%) | 10 (27) | 3 (27.3) |  |
| Abdominoperineal resection, n (%) | 8 (21.6) | 3 (27.3) | 0.6972 |
| Anastomosis technique, n (%) |  | | |
| Mechanical (stapled) | 27 (73) | 8 (72.7) | 1.0000 |
| Manual | 2 (5.4) | 0 |  |
| Liver surgery, n (%) |  | | |
| - Minor hepatectomy | 13 (35.1) | 11 (100) | **0.0002** |
| - Major hepatectomy | 24 (64.9) | 0 |  |
| - Radiofrequency ablation associated | 4 (10.8) | 3 (27.2) | 0.3271 |
| Duration of Pringle maneuver (minutes), median ± IQR | 28 (25 – 45) | 41 (32 – 75) | 0.0564 |
| Diverting stoma, n (%) | 17 (45.9) | 4 (36.4) | 0.7334 |
| Delay between first and second surgery (months), median ± IQR | 6 (4 – 8) | 6 (4 – 8) | 0.6192 |
| Post-operative liver embolization | 2 (5.4) | 0 | 1.0000 |
| Intra-operative portal ligation and/or embolization | 5 (13.5) | 0 | 0.5757 |

IQR : interquartile range

Major hepatectomy : resection of 3 segments or more

Hybrid : laparoscopy and laparotomy
