# Supplementary material for: Simultaneous or staged resection for synchronous liver metastasis and primary rectal cancer: a propensity score matching analysis
Source: BMC Gastroenterol. 2022 Apr 21;22:201. doi: 10.1186/s12876-022-02250-9 (PMC9026992; doi:10.1186/s12876-022-02250-9)
Supplement: Supplementary file 3 — Additional file 3. Table S2. Pathology results of the 48 patients with rectal cancer and synchronous liver metastases undergoing staged surgery according to their management. [file 12876_2022_2250_MOESM3_ESM.docx]

Table S2: Pathology results of the 48 patients with rectal cancer and synchronous liver metastases undergoing staged surgery according to their management

|  | Rectum first | Liver First | P |
| --- | --- | --- | --- |
|  |  |  |  |
| N (%) | 37 | 11 |  |
| Rectal pT stage, n (%) |  |  |  |
| - pT0 - pT1 - pT2 - pT3 - pT4 | 2 (5.4)  1 (2.7)  5 (13.5)  25 (67.6)  4 (10.8) | 1 (9.1)  1 (9.1)  0  7 (63.6)  2 (18.2) | `0.5765 |
| Rectal pN stage, n (%) |  |  |  |
| - pN0 - pN1 - pN2 | 12 (32.5)  16 (43.2)  9 (24.3) | 3 (27.3)  5 (45.4)  3 (27.3) | 0.7458  0.9388 |
| Number of lymph nodes harvested, median ± IQR | 25 (19 – 39) | 21 (18 – 31) | 0.7148 |
| Number of lymph nodes metastasis, median ± IQR | 1 (0 – 4) | 1 (0 – 4) | 0.7577 |
| Number of liver metastasis, median ± IQR | 2 (2 – 4) | 3 (1 – 8) | 0.2843 |
| Liver resection margin positive, n (%) | 9 (24.3) | 4 (36.3) | 0.4302 |
| - R1 (contact with liver tissue)  - R1 (contact with vascular structure)  - R2 | 7/9 (77.8)  1/9 (11.1)  1/9 (11.1) | 3/4 (75)  0  1/4 (25) | 0.6722 |
| Rectal resection margin positive, n (%) | 4 (10.8) | 2 (18.2) | 0.5163 |
| - lateral margin  - distal margin | 2/4 (50)  2/4 (50) | 2/2 (100)  0 | 0.2207 |
| Percentage of liver metastasis necrosis, median ± IQR | 40 (24 – 66) | 60 (33 – 80) | 0.4217 |
| Liver fibrosis, n (%) | 13 (35.1) | 1 (9.1) | 0.0952 |
| Liver steatosis, n (%) | 13 (35.1) | 2 (18.2) | 0.2869 |
| Capillary obstruction syndrome, n (%) | 6 (16.2) | 3 (27.3) | 0.4094 |
| Largest size of liver metastasis (mm), median ± IQR | 35 (18 – 45) | 21 (16 – 35) | 0.7408 |

IQR : interquartile range
